# The diversity and ecological significance of microbial traits potentially involved in B_12_ biosynthesis in the global ocean

**DOI:** 10.1002/mlf2.12095

**Published:** 2023-12-26

**Authors:** Jiayin Zhou, Wei Qin, Xinda Lu, Yunfeng Yang, David Stahl, Nianzhi Jiao, Jizhong Zhou, Jihua Liu, Qichao Tu

**Affiliations:** ^1^ Institute of Marine Science and Technology Shandong University Qingdao China; ^2^ Joint Lab for Ocean Research and Education at Dalhousie University Shandong University and Xiamen University Qingdao China; ^3^ School of Biological Sciences University of Oklahoma Norman Oklahoma USA; ^4^ Department of Civil and Environmental Engineering Massachusetts Institute of Technology Cambridge Massachusetts USA; ^5^ State Key Joint Laboratory of Environment Simulation and Pollution Control, School of Environment Tsinghua University Beijing China; ^6^ Department of Civil and Environmental Engineering University of Washington Seattle Washington USA; ^7^ Institute of Marine Microbes and Ecospheres Xiamen University Xiamen China; ^8^ Earth and Environmental Sciences, Lawrence Berkeley National Laboratory Berkeley California USA; ^9^ Institute for Environmental Genomics, University of Oklahoma Norman Oklahoma USA; ^10^ School of Civil Engineering and Environmental Sciences, University of Oklahoma Norman Oklahoma USA; ^11^ School of Computer Sciences, University of Oklahoma Norman Oklahoma USA; ^12^ Present address: DermBiont Inc. Boston Massachusetts USA

**Keywords:** B_12_ biosynthesis, community assembly, functional genes, functional redundancy, ocean primary production

## Abstract

Cobalamin (B_12_), an essential nutrient and growth cofactor for many living organisms on Earth, can be fully synthesized only by selected prokaryotes in nature. Therefore, microbial communities related to B_12_ biosynthesis could serve as an example subsystem to disentangle the underlying ecological mechanisms balancing the function and taxonomic make‐up of complex functional assemblages. By anchoring microbial traits potentially involved in B_12_ biosynthesis, we depict the biogeographic patterns of B_12_ biosynthesis genes and the taxa harboring them in the global ocean, despite the limitations of detecting de novo B_12_ synthesizers via metagenomes alone. Both the taxonomic and functional composition of B_12_ biosynthesis genes were strongly shaped by depth, differentiating the epipelagic zones from the mesopelagic layers. Functional genes related to B_12_ biosynthesis were relatively stably distributed across different oceans, but the taxa harboring them varied considerably, showing clear functional redundancy among microbial systems. Microbial taxa carrying B_12_ biosynthesis genes in the surface water were influenced by environmental factors such as temperature, oxygen, and nitrate. However, the composition of functional genes was only weakly associated with these environmental factors. Null model analyses demonstrated that determinism governed the variations in B_12_ biosynthesis genes, whereas a higher degree of stochasticity was associated with taxonomic variations. Significant associations were observed between the chlorophyll *a* concentration and B_12_ biosynthesis, confirming its importance in primary production in the global ocean. The results of this study reveal an essential ecological mechanism governing the assembly of microbes in nature: the environment selects for function rather than taxonomy; functional redundancy underlies stochastic community assembly.

## Impact statement

A central question in ecology is how a galaxy of microbial taxa is assembled and distributed across space and through time, executing essential ecosystem functions. By anchoring microbial functional traits potentially involved in B_12_ biosynthesis and their carrying microbial taxa in the global ocean, this study addresses essential ecological questions from functional and taxonomic angles. Integrating multiple lines of evidence, we show that the ecosystem selects functional traits rather than taxonomic groups, and functional redundancy underlies stochastic taxonomic community assembly. Also, microbial communities potentially involved in B_12_ biosynthesis are significantly associated with chlorophyll *a* concentration, demonstrating their importance in global ocean primary production. This study provides valuable mechanistic insights into the complex microbial community assembly in ecosystems.

## INTRODUCTION

As the home to a galaxy of life forms^1^, the global ocean accounts for roughly 97% of the water on Earth, provides 50% of the oxygen and plays an irreplaceable role in impacting the global climate^2^, ^3^. Microbial communities, the unseen majority^4^, are of fundamental importance in maintaining the functionality and stability of the global ocean's ecosystems. They not only drive the global biogeochemical cycling of various nutrients and elements and maintain multiple functions in the ecosystem^5^, ^6^, but also provide essential nutrients to other organisms, including both prokaryotes and eukaryotes^7^. One such example is B_12_, an essential nutrient and growth cofactor that is utilized extensively by prokaryotes and eukaryotes for numerous metabolic functions^8^, ^9^, ^10^, ^11^. In natural ecosystems, B_12_ biosynthesis is energetically extremely expensive, which imposes a high metabolic burden upon B_12_ producers^12^. Only a small cohort of prokaryotes holds the genetic potential to accomplish such a complex process, while the others have to rely on exogenous supply, forming the “corrinoid economy”^13^. Therefore, B_12_ auxotrophs may establish close mutualistic interactions with B_12_ producers, offsetting the cost of B_12_ biosynthesis to ensure sustainable sources^14^. Such interactive relationships have significant impacts on the composition and structure of marine microbial communities. Two distinct pools of B_12_ analogs were found in the ocean: the B_12_ pool produced by a few prokaryotes such as *Thaumarchaeota* and alpha‐/gamma‐proteobacterial lineages (e.g., *Rhodobacterales*, *Rhizobiales*, and most members of the *Rickettsiales*)^7^, ^11^, ^14^, ^15^, and the pseudocobalamin pool produced by *Cyanobacteria* as representatives^11^, ^14^. In recent years, the importance of B_12_ has been widely recognized. It influences the growth rate of phytoplankton in the ocean^16^, affects the size and diversity of microbial communities in terrestrial ecosystems^17^, and affects the health status of gut microbes in the human intestinal system^18^, ^19^. In addition, the availability of B_12_ has critical impacts on both cellular‐level metabolic processes (e.g., methionine synthesis)^20^ and system‐level biogeochemical cycling (e.g., photosynthesis, aerobic nitrogen cycle)^7^, ^21^, ^22^. As one of the highly limited nutrients and growth factors controlled by a minority of microbes, B_12_ can be considered as a “hard currency” in the global ocean ecosystem.

Several studies have focused on the importance of marine B_12_ biosynthesis in recent years. For example, most of the eukaryotic phytoplanktons in the surface ocean are B_12_ auxotrophs^9^, and their growth rate can be limited by B_12_ availability, which further affects their primary productivity. In addition, stoichiometric studies of diatoms in the Subarctic Pacific showed that the carbon:phosphorus (C:P) ratios of B_12_‐limited cells are significantly lower in comparison with B_12_‐replete cells^23^. This phenomenon is becoming more pronounced with the significantly increased partial pressure of CO_2_ caused by anthropogenic activities and global climate change. For example, the C:P ratio gap between B_12_‐replete and B_12_‐limited cells was found to gradually widen as the carbon dioxide partial pressure (pCO_2_) increased, reaching about 40% at 670 ppm pCO_2_ ^23^. Recent studies have also demonstrated that the growth rate and primary productivity of phytoplankton are affected by B_12_ availability^22^, ^23^, ^24^. Although B_12_ is of critical importance, the diversity, distribution, and underlying ecological mechanisms shaping the patterns of microbial communities involved in B_12_ biosynthesis in the global ocean remain largely unexplored. Studies focused on this topic will not only provide a clearer understanding of this subset of prokaryotes in the global ocean but also shed light on the consequential global ocean ecosystem function. Importantly, the *Tara* Oceans Expedition^25^, ^26^, ^27^ provides a valuable resource that includes comprehensive data sets at the global scale, covering a total of eight ocean regions and three ocean depth ranges, making it possible to investigate the global patterns of various microbial (sub)communities, including the microbial taxa related with B_12_ biosynthesis.

In this study, by utilizing the *Tara* Oceans shotgun metagenome sequencing data sets, we surveyed the diversity patterns and ecological importance of microbial traits (functional genes and the corresponding taxonomic groups) potentially involved in B_12_ biosynthesis in the global ocean ecosystem. Community‐level investigations were mainly performed because of the limitations of identifying de novo B_12_ synthesizers from metagenomes alone. The following essential ecological questions were addressed: (i) How are B_12_ biosynthesis traits distributed globally? (ii) What ecological mechanism drives and maintains the diversity patterns of B_12_ biosynthesis traits? (iii) How do microbial B_12_ biosynthesis traits contribute to the functions of the global ocean ecosystem, for example, the ocean's primary production? Because of their critical importance to the global ocean, microbial functional genes involved in B_12_ biosynthesis were expected to show a relatively stable abundance and distribution across the global ocean. However, because of functional redundancy among microbial systems^28^, the microbial taxonomic groups carrying them may vary across different oceanic regions and depths. Determinism, therefore, should be mainly responsible for the diversity patterns of functional traits. However, compared with functional traits, microbial taxonomic groups would be more strongly influenced by stochastic processes, due to functional redundancy among microbial systems. Our results support the above hypotheses and show that B_12_ biosynthesis traits are significantly associated with the chlorophyll *a* concentration, confirming their important role in primary production in the global ocean.

## RESULTS

### Overall diversity of potential B_12_ biosynthesis traits in the global ocean

**Figure 1:**
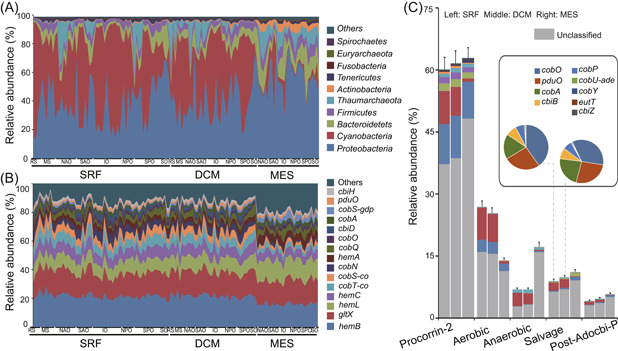
Composition of microbial taxonomic groups and functional traits related to B_12_ biosynthesis in the global ocean. (A) Composition of microbial taxa carrying B_12_ biosynthesis genes across different samples. (B) Composition of microbial functional traits potentially involved in B_12_ biosynthesis across different samples. (C) Relative abundance of microbial phyla carrying genes in different B_12_ biosynthesis pathways and different ocean layers. Pie charts show the relative abundance of functional traits related to the salvage pathway in the epipelagic zone. The same scaling color code is used in (A) and the stacked bar chart in (C). The figure shows major microbial taxa and functional traits. DCM, deep chlorophyll maximum layer; MES, mesopelagic zone; SRF, surface water layer.

Only a small fraction of prokaryotes can fully synthesize B_12_ ^7^, ^15^ because of the multiple enzymatic steps involved (Figure S1). By applying VB_12_Path^29^ to the *Tara* Oceans shotgun metagenome data set, an average of 0.2% reads per sample were identified to encode gene families potentially involved in B_12_ biosynthesis pathways. Consistent with the result of the *Tara* Oceans study that microbial communities significantly differ between the mesopelagic layer (MES) and the epipelagic zones^26^, the same pattern was observed for microbial taxa carrying B_12_ biosynthesis genes. Compared with microbial communities in the epipelagic zone, those potentially involved in B_12_ biosynthesis in the MES showed significantly higher taxonomic and functional diversity as well as dramatically different composition (Figures 1A, S2–S4 and Table S1). Surprisingly, the evenness of B_12_ biosynthesis functional traits and their carrying taxa were negatively correlated, leading to a negative correlation between community diversity (Shannon–Wiener index) (Figure S5). The negative correlation is likely because only a small fraction of microbial taxa carry a (nearly) full set of gene families involved in B_12_ biosynthesis; therefore, the even distribution of microbial taxa resulted in an uneven distribution of functional traits.

At the pathway level, microbial functional traits potentially involved in precorrin‐2 synthesis (63.84%) and aerobic B_12_ biosynthesis (24.48%) pathways exhibited the highest relative abundance in the *Tara* Oceans samples, while anaerobic (9.26%) and post‐Adocbi‐P (4.87%) pathways were less abundant (Figure 1C). At the functional gene level, gene families related to the aerobic B_12_ biosynthesis pathway were generally more abundant in the epipelagic zones, while the ones related to the anaerobic pathway were more abundant in the MES (Figure 1C and Table S4). Most importantly, consistent with our expectations, the relative abundance of functional genes related to B_12_ biosynthesis was relatively stable in the global ocean (Figure 1B), while the taxonomic composition was highly variable. This pattern was observed for microbial communities sampled from different depth intervals and oceanic regions (Figure 1A). These results pinpointed an essential microbial ecological discipline that taxonomically highly varied microbial communities still executed similar ecosystem functions.

### Microbial taxa carrying B_12_ biosynthesis genes in the global ocean

Among the identified microbial taxa containing B_12_ biosynthesis genes, *Proteobacteria* were abundantly detected in all samples, whereas *Cyanobacteria* were dominant in the epipelagic zones and dramatically depleted in the MES. Compared with their abundance in the epipelagic zones, *Thaumarchaeota* was significantly enriched in the MES, and harbored genes related to the anaerobic pathway of B_12_ biosynthesis (specifically, nine *cbi* genes were detected) (Figure 1A,C and Table S3). Different modules of the B_12_ biosynthesis pathway were featured by different microbial taxonomic groups (Figure 1C). This was especially evident for taxa in the MES. Microbial taxa belonging to *Thaumarchaeaota* and *Bacteroidetes* were, respectively, dominantly observed with genes belonging to anaerobic and salvage pathways. This result agreed with those of previous studies suggesting that B_12_ in the surface ocean may be primarily the result of de novo synthesis by heterotrophic bacteria or via modification of pseudocobalamin produced by *Cyanobacteria*, whereas *Thaumarchaeota* may be the major B_12_ producers at depth^14^. Despite the high abundance of *Bacteroidetes* in the MES, studies have shown that only 0.6% of *Bacteroidetes* harbor complete B_12_ synthesis pathways^15^. Gene families (e.g., *cobO*, *pduO*, and *cobA*) belonging to the salvage pathway were dominantly carried by *Cyanobacteria*, more specifically *Prochlorococcus* (Figure 1C and Table S2). A quick BLAST searching these gene families against *Prochlorococcus* genomes in the NCBI database suggested that these gene families are widespread among *Prochlorococcus* (data not shown). While *Cyanobacteria* are generally pseudocobalamin synthesizers^30^, the fact that *Prochlorococcus* carries gene families involved in the salvage pathway indicated the potential of this genus to remodel B_12_ precursors/analogs under certain conditions. Notably, a recent genomic study also detected gene families involved in the salvage pathway in *Synechococcus* genomes, possibly due to horizontal gene transfer events or loss of function (of de novo B_12_ biosynthesis) during evolution^31^. In addition, a high portion of microbial taxa carrying B_12_ biosynthesis genes belonged to unclassified taxonomic groups, especially in the MES, suggesting that much remains to be further explored for the B_12_ biosynthesis genes and the taxa that harbor them in the deep ocean.

Microbial taxa potentially involved in B_12_ biosynthesis in the global ocean were further investigated (Table S2) by selecting the putative key B_12_ synthesis gene families identified in previous investigations^7^, ^30^. Although B_12_ biosynthesis genes were detected in many microbial taxa, those carrying complete de novo B_12_ biosynthesis pathways were rarely found, possibly due to inadequate sequencing depth to detect these genes and/or because of the rarity of microbial taxa containing complete B_12_ biosynthesis pathways. Overall, microbial species including *Prochlorococcus marinus*, Candidatus *Nitrosopelagicus brevis*, Candidatus *Nitrosomarinus catalina*, and *Synechococcus* sp. CC9902 were the taxa carrying a large number of key B_12_ biosynthesis gene families. Although B_12_ biosynthesis genes have been detected in some microbial families (e.g., *Synechococcaceae*, *Prochlorococcaceae*, and *Pelagibacteraceae*), these taxa are considered to be auxotrophic because they lack the gene families necessary for 5,6‐dimethylbenzimidazole (DMB) synthesis, such as *bluB* ^32^, ^33^, and for DMB activation, such as *cobT* ^30^. For example, members of the genus *Synechococcus* contain many genes belonging to B_12_ biosynthesis pathways but lack key genes for DMB synthesis (Table S2) and have been shown to be B_12_ auxotrophic^30^. Therefore, detection of B_12_ biosynthesis genes in microbial taxa does not necessarily imply the capacity of de novo biosynthesis of this cofactor. Further experimental evidence is required to validate such a capacity. These results also highlighted the challenges in identifying potential B_12_ synthesizers using metagenomic approaches, on the basis that the majority of microbial taxa were unknown and metagenomic recovery of rare microbial taxa was almost impossible.

### Latitudinal diversity patterns and distance–decay relationships (DDR)

**Figure 2:**
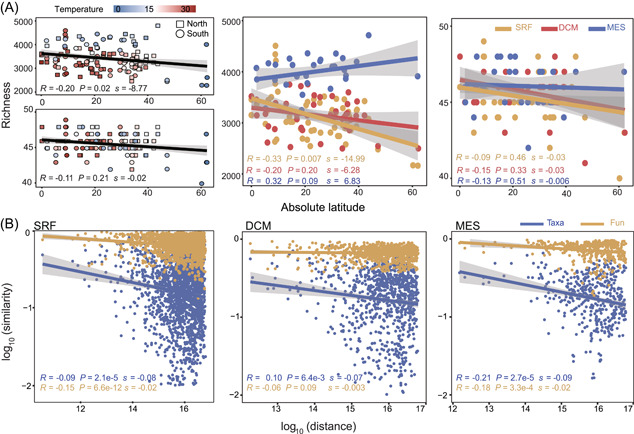
Biogeographic patterns of potential B_12_ biosynthesis traits in the global ocean. (A) Latitudinal diversity gradient (LDG) patterns for B_12_ biosynthesis traits in the global ocean. (B) Distance–decay relationship (DDR) for B_12_ biosynthesis traits in the global ocean. Patterns of taxonomic groups and functional traits were investigated. Fun, functional composition; Taxa, taxonomic composition.

We also investigated whether microbial communities potentially involved in B_12_ biosynthesis followed typical biogeographic patterns such as a latitudinal diversity gradient (LDG) and/or DDR, which are well‐recognized ecological patterns for both microbial and macrobial communities^34^, ^35^. Discordant patterns between the composition of microbial taxonomic groups and the composition of functional genes were observed in this study (Figure 1A,B). Although B_12_ biosynthesis serves as an essential ecosystem function and shall be stably maintained in the global ocean, the microbial taxa carrying these functional traits are influenced by various environmental conditions. We expected clear LDG and DDR patterns for microbial taxa carrying B_12_ biosynthesis genes, but weaker or even nonexistent patterns for the functional genes. Consistent with our expectation, a weak LDG pattern was detected for the functional genes at the surface water layer (SRF) (*P* = 0.02), but not at the deep chlorophyll maximum layer (DCM) and the MES. No significant DDR pattern was detected for B_12_ biosynthesis genes at all three pelagic zones. For microbial taxa carrying B_12_ biosynthesis genes, a strong LDG pattern was observed at the SRF (*P* = 0.007), whereas DDR was observed at all three pelagic zones (*P* ≤ 0.001) (Figure 2A,B). Such distinct biogeographic patterns of functional genes and taxonomic groups again pointed to an essential microbial ecology principle, that is, microbial functional genes executing essential ecosystem functions are prevalently distributed, whereas their carrying microbial taxa may vary dramatically.

### Environmental factors associated with variations in potential B_12_ biosynthesis traits

Next, we investigated the associations between B_12_ biosynthesis traits and environmental factors (Figure S6). Since both the functional and taxonomic compositions of B_12_ biosynthesis genes dramatically differ by depth, the associations with geo‐environmental factors were analyzed for a given range of water depths, thereby eliminating the effects of depth and depth‐correlated environmental factors. As a result, weakened effects of environmental factors on the taxonomic compositions were observed from the SRF to the MES. In the SRF, the concentrations of dissolved oxygen and nitrate availability were significantly associated with the taxonomic composition. Such effects, however, were gradually diminished in the DCM and MES layers. Interestingly, no significant associations were detected between environmental factors and the functional composition of B_12_ biosynthesis genes in all three oceanic layers, suggesting that environmental conditions mainly affected the taxonomic composition.

The associations between environmental factors and community diversity were also investigated. Significant associations between environmental factors and community diversity could be observed (Figure S7A). However, such effects were weakened or even diminished when looking at individual pelagic zones (Figure S7B–D), suggesting that depth differences from the SRF to the MES and their correlations with environmental factors were mainly responsible for such “pseudo‐associations.” Surprisingly, the effects of temperature on the B_12_ biosynthesis functional trait diversity differed dramatically among oceanic layers. Temperature was positively associated with functional gene diversity in the epipelagic layers (Figure S7B,C), but negatively in the MES (Figure S7D), leading to a nonsignificant association across the whole upper ocean (Figure S7A). Such opposite patterns were also observed for other environmental factors such as oxygen, nitrite and nitrate concentration (NO_2_NO_3_), and nitrate, although some of them were not statistically significant (*P* ≥ 0.05).

### Ecological mechanisms governing the assembly of B_12_ biosynthesis traits

**Figure 3:**
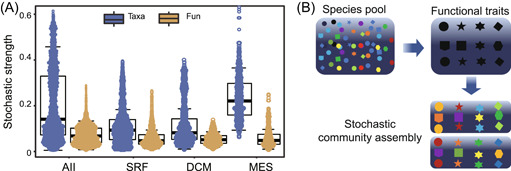
Mechanisms governing assembly of B_12_ biosynthesis traits in the ocean ecosystem. (A) Stochasticity of community assembly as revealed by null model analysis. (B) Ecological model explaining community assembly of microbial functional groups in the ocean ecosystem. According to the model, the environment selects microbial functional traits rather than taxonomic groups, and functional redundancy underlies stochastic community assembly. In the ecological model, different colors represent different microbial taxa, whereas different shapes represent different functional traits.

Considering the critical roles that B_12_ plays in the ecosystem, we expected that the assembly of microbial functional traits would be highly deterministic. To examine this hypothesis, we quantified the relative importance of deterministic and stochastic processes in governing the assembly of functional traits potentially involved in B_12_ biosynthesis. In this study, the null model analysis was employed to characterize the ratio of stochasticity to determinism by comparing the observed and null model community β‐diversity (Figure 3A). Consistent with our hypothetical expectations, the stochastic ratio suggested that both the assembly of microbial functional genes and their carrying taxa were highly deterministic. Compared with the functional traits, the taxonomic groups had higher stochastic ratios, especially in the MES, suggesting that the assembly of taxonomic groups was more stochastic than functional traits. Such patterns of stochastic ratios between functional traits and taxonomic groups were consistent in different oceanic layers.

We hypothesized that deterministic factors should govern the assembly of microbial functional traits and that the assembly of microbial taxa shall be relatively more stochastic than functional traits. All the results described above, for example, the stable distribution of functional traits versus highly varied taxonomic groups (Figure 1A,B), stronger biogeographic patterns for taxonomic groups than for functional traits (Figures S6 and S7), and the relative importance of deterministic and stochastic processes (Figure 3A), provided evidence to support our hypotheses for community assembly of B_12_ biosynthesis traits. Integrating all lines of evidence, we proposed a functional trait‐based ecological model to explain complex microbial community assembly in natural ecosystems (Figure 3B). Variations in geo‐environmental factors such as depth, temperature, and oxygen form multiple ecological niches in the oceanic ecosystem (e.g., the epipelagic zones and the MES). Microorganisms capable of living in these ecological niches comprise the species pools. To maintain fundamental ecosystem functions, microorganisms carrying essential functional traits are selected. Therefore, it is the function, rather than taxonomy that the environment truly selects^36^. However, owing to functional redundancy among microbial systems^28^, different taxonomic groups carry the same functional traits. Meanwhile, stochastic processes such as drift and dispersal are associated with microbial taxa. Stochastic community assembly occurs simultaneously with the selection of functional traits. As a result, varied taxonomic compositions come with comparable combinations of functional traits, as observed in multiple ecosystems^37^, ^38^, ^39^. For microbial traits potentially involved in B_12_ biosynthesis, both taxonomic groups and functional traits were governed by deterministic processes, and functional redundancy of microbial taxonomic groups led to higher stochasticity in community assembly.

### Ecological importance of potential B_12_ biosynthesis traits in the global ocean

**Figure 4:**
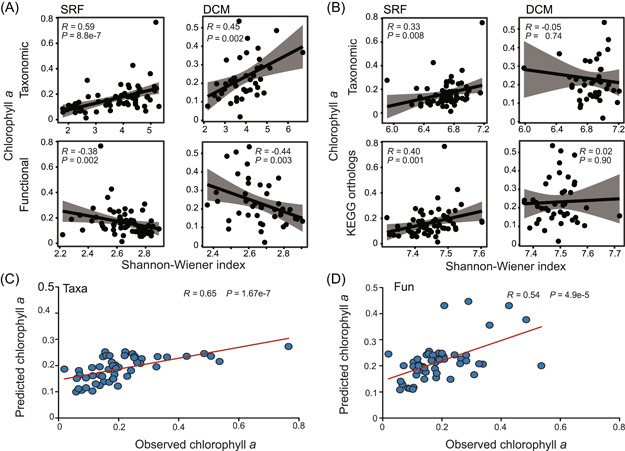
Association between microbial community diversity and chlorophyll *a* concentration in the global ocean. (A) Association (Spearman's *ρ*) between B_12_ biosynthesis trait diversity (taxonomic and functional trait) and chlorophyll *a* concentration. (B) Association (Spearman's *ρ*) between overall prokaryotic community diversity (taxonomic and KEGG orthologous groups) and chlorophyll *a* concentrations. (C) Chlorophyll *a* concentrations predicted from microbial taxa carrying B_12_ biosynthesis genes. (D) Chlorophyll *a* concentrations predicted from B_12_ biosynthesis functional trait profiles. KEGG, Kyoto Encyclopedia of Genes and Genomes.

Finally, we investigated the ecological roles of potential B_12_ biosynthesis traits in the oceanic ecosystem, such as their effects on B_12_‐dependent microorganisms and their contribution to the ocean's primary production^7^, ^9^, ^14^, ^24^. To investigate whether B_12_ biosynthesis traits are potentially associated with B_12_‐dependent microbial communities and global ocean primary productivity, we investigated the associations between the community diversity of B_12_ biosynthesis traits and the relative abundance of the metH gene family (encoding B_12_‐dependent methionine synthase) and the chlorophyll *a* concentration. First, a significant association was observed between the relative abundance of the metH gene family and B_12_ biosynthesis trait diversity (Figure S8), confirming the importance of B_12_ biosynthesizing‐members to B_12_‐dependent members in the oceanic ecosystem. Second, the concentration of chlorophyll *a* in the epipelagic zone was also significantly associated with B_12_ biosynthesis trait diversity (*P* ≤ 0.005) (Figure 4A). Notably, the concentration of chlorophyll *a* was positively correlated with the taxonomic diversity but negatively correlated with functional gene diversity of B_12_ biosynthesis traits. Such an opposite pattern was attributed to the negative correlation between the evenness of B_12_ biosynthesis genes and the taxa harboring them (Figure S8). To exclude the potential influence of the whole microbial community and further confirm the significant correlation between the chlorophyll *a* concentration and B_12_ biosynthesis traits, we also evaluated the association between the chlorophyll *a* concentration and the diversity of the prokaryotic community (taxonomic and Kyoto Encyclopedia of Genes and Genomes [KEGG] orthologous groups). The strength of the association between the chlorophyll *a* concentration and prokaryotic community diversity was either nonsignificant or much weaker than that of the association with B_12_ biosynthesis traits (Figure 4B). Finally, a random forest machine learning approach was employed to further verify the importance of B_12_ biosynthesis traits by predicting the chlorophyll *a* concentration from B_12_ community profiles. The results demonstrated that both the taxonomic and functional profiles of B_12_ biosynthesis traits can well predict the concentration of chlorophyll *a* in the ocean (Figure 4C,D). This also held true when using SRF microbial data as the training data set to predict the chlorophyll *a* concentration in the DCM layer, or vice versa (Figure S9).

## DISCUSSION

Focusing on “who is doing what, where, and how?” this study investigated the ecological mechanisms driving the patterns of diversity of microbial traits potentially involved in B_12_ biosynthesis and their ecological importance in the global ocean. Because of the limitations of the rarity of the targeted microbial taxa and current technologies, it was difficult to confidently infer specific de novo B_12_ synthesizers. Therefore, community‐level investigations were performed in this study. Similar to what has been observed for the global ocean microbiome^26^, both the taxonomic and functional gene composition related to B_12_ biosynthesis differed by depth instead of oceanic regions. Multiple factors such as depth, light, temperature, and other associated environmental factors are responsible for such patterns. This suggests that there are completely different niche preferences of B_12_ biosynthesis traits in different oceanic layers. We also noticed that the evenness of B_12_ biosynthesis genes and their carrying taxa were negatively correlated, suggesting that an even distribution of microbial taxa may not lead to an even distribution of functional traits. This negative correlation is due to the fact that only a small fraction of microbial taxa contain (near) complete B_12_ biosynthesis pathways in their genomes, and an even distribution of microbial taxa does not reflect even functional traits.

Microbial taxa carrying B_12_ biosynthesis genes in the ocean ecosystem were also investigated at a refined taxonomic resolution. However, limited information was gained in this analysis. First, the taxonomy of the majority of B_12_ biosynthesis genes remained unclassified, even when searched against taxonomic databases built from the most recent NCBI database. This was especially the case for microbial taxa in the MES. Such a shortage of taxonomic information is mainly because of the limitations of current genomic databases^40^, the fact that the majority of microbial taxa in nature remain uncultured^41^, and the potential limitations of read‐based analyses. This result also suggests that there is still much to learn about this tiny group of microorganisms on Earth, especially in the deep ocean. Second, consistent with our current knowledge^14^, only a few microbial genera in the ocean were found to have the potential to synthesize B_12_ de novo, judging by the gene families linked to the microbial taxa. However, comparative genomic analyses of sequenced microbial genomes from NCBI RefSeq suggest that 37% of prokaryotic microbial species have the potential to biosynthesize cobamides de novo, although complete pathways are not always detected^15^. Among these, 57% of *Actinobacteria* are predicted to biosynthesize cobamides, whereas only 0.6% of *Bacteroidetes* have the complete pathway^15^. Such inconsistencies between metagenomic and genomic studies are due to the rarity and unknown properties of de novo B_12_ synthesizers in the ocean and because current sequencing technologies and depth may not capture them well. Third, identifying de novo B_12_ synthesizers is challenging and requires further attention. *Rhodobacteraceae*, *Rhizobiales*, and a subset of *Cyanobacteria* were found to be the most important candidates as B_12_ prototrophs in neritic ecosystems in metatranscriptomic and metaproteomic analyses^42^. However, one needs to be aware that the lower ligand must be DMB to produce B_12_ and not pseudocobalamin. Perhaps judgment based on key genes related to the synthesis and activation of DMB, for example, *bluB* ^32^, ^33^ and *cobT* ^30^, is also an option. *Cyanobacteria* strains release pseudo‐B_12_ into the media at a high rate, so it has been speculated that *Cyanobacteria* may be the main providers of (pseudo‐)B_12_ in algal metabolism^43^. Similarly, genes potentially involved in B_12_ biosynthesis have been frequently detected in cyanobacterial genera such as *Synechococcus* and *Prochlorococcus*, which may only produce pseudocobalamin because adenine is the lower ligand instead of DMB, consistent with previous studies^7^, ^11^, ^14^. In certain cases, microbial taxa (e.g., *Dehalococcoides mccartyi* strain 195, *Chlamydomonas reinhardtii*) may remodel nonfunctional cobamides (e.g., pseudocobalamin) to B_12_ under suitable environmental conditions such as at the presence of DMB or its intermediate α‐ribazole^11^, ^30^, ^44^. Interestingly, *bluB* and *cobT* were detected from *P. marinus* at a high taxonomic level (Table S2), and previous studies also mentioned that the *P. marinus* SS120 genome may encode the full set of enzymes in the heme B_12_ biosynthetic pathway^45^. In the marine ecosystem, *Rhodobacterales* are the major alphaproteobacterial B_12_ producers, but we did not detect *bluB* from them (e.g., *Epibacterium mobile*). Therefore, even if these key B_12_ biosynthesis gene families are detected, further experimental validation is needed to confirm their function in the ecosystem.

This study also revealed important implications in terms of the ecological roles that B_12_ biosynthesis traits play in the oceanic ecosystem. Previous studies have suggested that eukaryotic phytoplankton in the surface ocean are B_12_ auxotrophs^9^, ^30^, and their growth rate may be limited by B_12_ availability, further affecting ocean primary productivity^16^, ^24^, ^46^, ^47^. The requirements of these eukaryotic algae for B_12_ are primarily mediated by methionine synthase^9^, ^48^, a key enzyme in cellular one‐carbon metabolism responsible for catalyzing the conversion of homocysteine and 5‐methyl‐tetrahydrofolate to tetrahydrofolate and methionine^49^, ^50^. Although B_12_‐independent methionine synthase (MetE) and B_12_‐dependent methionine synthase (MetH) are capable of completing this reaction^9^, ^48^, MetE is approximately 100‐fold less catalytically efficient than MetH^51^, and this inefficiency further results in an approximately 30‐ to 40‐fold increase in nitrogen and zinc requirements for MetE compared with MetH^52^. Consistent with previous studies, we detected significant correlations between B_12_ biosynthesis traits and *metH* encoding B_12_‐dependent methionine synthase, and between B_12_ biosynthesis traits and the chlorophyll *a* concentration. This suggests that B_12_ biosynthesis traits exert strong effects on the chlorophyll *a* concentration, demonstrating the importance of this microbial group to the global ocean's primary production.

Our results reveal the diversity patterns of B_12_ biosynthesis traits in the oceanic ecosystem. The microbial subcommunities also served as an example to reveal an intriguing functional trait‐based ecological mechanism explaining complex microbial community assembly in nature. Both deterministic and stochastic processes govern microbial community assembly, and a major question is which one is more important^53^, ^54^, ^55^. Considering that B_12_ biosynthesis is an essential ecosystem function and shall be stably maintained in the global ocean^7^, ^15^, ^56^, we speculated that strong determinism should govern the assembly of potential B_12_ biosynthesis traits. However, microbial communities are usually functionally redundant^28^, that is, multiple different microbial taxa may execute the same function. Similar to previous studies on the ocean's microbiome^26^, ^57^, ^58^, high functional redundancy was also observed in this study. A previous study suggested that the ecosystem tends to select microbial functional traits rather than taxonomic groups^36^. In addition, stochastic processes such as drift and dispersal are associated with microbial taxa^59^. As multiple microbial taxa carry the same functional traits, a certain degree of randomness is associated with microbial taxa in the ecosystem. Consistent with our expectations, higher stochasticity was observed in the assembly of microbial taxa than in functional traits. To summarize, the environment selects microbial functional traits rather than taxonomic groups^36^, and functional redundancy^28^ underlies stochastic microbial community assembly, thereby maintaining essential ecosystem function and stability^60^. In addition, we urge that mechanistic studies on microbial community ecology should not only focus on microbial taxonomic groups but also on the functional genes that they carry. Whenever possible, microbial functional genes and taxonomy should be equally considered in microbial systems.

In conclusion, using the B_12_ biosynthesis subsystem as an example, this study investigated the diversity, biogeographic patterns, and ecological drivers of this specific microbial functional group in the global ocean. Comparative analyses of the patterns of B_12_ biosynthesis genes and the microbial taxa that harbor them revealed an important microbial ecological mechanism, elucidating the relationship between natural ecosystems and complex microbial communities from the functional angle. Also, B_12_ biosynthesis traits were significantly associated with the chlorophyll *a* concentration, demonstrating the importance of this function in primary production in the global ocean. The results of this study provide valuable mechanistic insights into complex microbial community assemblies in natural ecosystems.

## MATERIALS AND METHODS

### *Tara* Oceans shotgun metagenomes and geo‐environmental factors

A total of 359 shotgun metagenomes targeting 138 samples covering three oceanic layers, including the SRF (5–10 m), DCM (17–180 m), and MES (250–1000 m), were downloaded from the European Bioinformatics Institute (EBI) repository under project ID ERP001736^26^. Forward and reverse reads were merged into longer sequences by the program PEAR (version 0.9.6, ‐q 30)^61^. An average of 208,881,758 merged reads per sample were obtained. Geo‐environmental factors, the overall taxonomical profiles, and KEGG orthologous group profiles associated with the shotgun metagenome data were downloaded from http://ocean-microbiome.embl.de/companion.html. Metadata for chlorophyll *a* concentrations in these *Tara* Oceans samples were obtained from the ZENODO website under the record number 7739198 (https://zenodo.org/record/7739198) according to a previous study^62^_._

### Metagenomic profiling of marine functional genes potentially involved in B_12_ biosynthesis

To keep the fidelity of taxonomic and functional profiles and get more usable information from the metagenomic data set^63^, read‐based analysis was performed. Considering the accuracy of gene definition and computational efficiency, VB_12_Path^29^, a specific functional gene database for metagenomic profiling of gene families involved in B_12_ biosynthesis pathways, was employed. Although this database is relatively small, both targeted gene families and their homologs from large public databases (e.g., KEGG, eggNOG, and COG) are integrated, minimizing false positive assignments. Briefly, merged metagenomic reads were searched against VB_12_Path. A total of 54 gene families involved in five modules of B_12_ biosynthesis pathway as previously described^29^, including precorrin‐2 synthesis processes, aerobic pathway, anaerobic pathway, salvage and remodeling pathway, and post‐Adocbi‐P pathway, are targeted in the database. The program DIAMOND (version 0.9.25, option: ‐k 1 ‐e 0.0001)^64^ was used to search nucleotide sequences against VB_12_Path using the blastx mode. Sequences matching VB_12_Path were retrieved to generate functional profiles targeting gene families involved in marine B_12_ biosynthesis using the PERL script provided in VB_12_Path. To minimize bias associated with sequence number variations across different samples, rarefaction was applied to each metagenome by a random subsampling effort of 100,000,000 sequences. Four samples were excluded from further analysis due to insufficient sequences.

To obtain taxonomic profiles for microbial taxa carrying B_12_ biosynthesis genes, merged metagenomic sequences belonging to targeted gene families in VB_12_Path were extracted by the seqtk program (https://github.com/lh3/seqtk). Extracted sequences were then subjected to taxonomic assignment by Kraken2^65^. A standard Kraken2 database was built locally based on the most recent NCBI database at the time this study was carried out. Taxonomic profiles were generated at multiple taxonomic levels based on the Kraken2 report files. After obtaining the functional and taxonomic profiles, the Kruskal–Wallis test was conducted to estimate statistical differences in relative abundances of potential B_12_ biosynthesis taxonomic groups and functional traits between the epipelagic (SRF/DCM) zone and MES. The false discovery rate approach was employed to adjust the *P* value to control for false positives using the “stats” package in R. All gene families of the B_12_ biosynthetic pathway, and the microbial taxa containing B_12_ biosynthetic gene families are collectively referred to as B_12_ biosynthesis traits in the context.

### Diversity indices

Various diversity indices were calculated by the “vegan” package^66^ in R (software version 4.0.3). Specifically, the richness, Shannon–Wiener index, and Pielou's evenness index were calculated for within‐sample diversity, that is, alpha diversity. The Bray–Curtis dissimilarity was calculated to represent between sample diversity, that is, community dissimilarity or beta diversity. The complement of community dissimilarity (1−dissimilarity) was calculated to quantify community similarity. Both within‐sample and between‐sample diversity indices were calculated for functional and taxonomic profiles. Compositional variance among samples in different layers and oceans, as well as epipelagic zone and MES, was calculated using Bray–Curtis dissimilarities and explored by principal coordinates analysis (PCoA), of which the first two axes were extracted for visualization. Three different nonparametric analyses, including permutational multivariate analysis of variance, analysis of similarity, and multiresponse permutation procedure, were performed to evaluate the statistical significance of compositional variations among SRF, DCM, and MES layers.

### LDG and DDR

Two major biogeographic patterns, including the LDG and DDR, were analyzed to investigate the diversity trend of B_12_ biosynthesis traits. For LDG, the relationship between community richness (species and functional traits) and absolute latitude was analyzed. For DDR, the relationship between community similarity and geographic distance was analyzed. The geographic distance between different samples was calculated by the Vincenty Ellipsoid formula based on the latitude and longitude coordinates using the “geosphere” package in R^67^. Community similarity values (Bray–Curtis indices) were obtained by subtracting community dissimilarity from 1. For DDR analyses, both the geographic distance and community similarity values were logarithmically transformed. For both LDG and DDR, linear regression analysis was carried out to visualize the diversity trendline. Values including correlation coefficients, slope, and significance *P* values were calculated. Analyses were performed for samples in three different layers.

### Correlating environmental factors with the diversity and composition of microbial communities

To identify the potential environmental factors shaping the variations of B_12_ community diversity and composition, the partial Mantel test was performed by correcting geographic distance. Bray–Curtis dissimilarity was selected to characterize the community distance for both taxonomic and functional trait profiles. The Euclidean distance method was used to characterize the distance between environmental factors. A permutation time of 9999 was set for the partial Mantel test. A total of 11 environmental variables were recruited, including latitude, longitude, depth, temperature, oxygen, mean nitrates concentration, NO_2_, nitrite and nitrate concentration (NO_2_NO_3_), phosphate (PO_4_), salinity, and silica (Si). To analyze the associations between environmental factors and community diversity, redundancy analysis was used to evaluate the collinearity between environmental variables and the taxonomic and functional trait composition. After excluding variables with high collinearity, a total of six geo‐environmental variables were retained, including depth, temperature, oxygen, nitrates, NO_2_NO_3_, and PO_4_. Then, linear regression analyses were conducted to investigate the relationships between each remaining individual environmental variable and community diversity (Shannon–Wiener index). Spearman's rank coefficient of correlation was calculated. All of the above statistical analyses were performed using the “vegan” package^66^ in R.

### Correlating *metH* gene abundance and chlorophyll *a* concentrations with B_12_ biosynthesis trait diversity

To disentangle the potential effects of B_12_ biosynthesis traits on B_12_‐dependent microbial communities and the ocean's primary productivity, the *metH* gene relative abundance and chlorophyll *a* concentration were correlated with the community diversity of B_12_ biosynthesis traits. Of these, *metH* gene was selected for its encoding of B_12_‐dependent methionine synthase, a pivotal enzyme of cellular one‐carbon metabolism and DNA synthesis^48^. Positive associations were expected between *metH* communities and B_12_ biosynthesis functional genes. Chlorophyll *a* was selected as a proxy for phytoplankton biomass to further approximate primary productivity. Linear regression analysis was used to explore the relationship between *metH* relative abundance, the chlorophyll *a* concentration, and B_12_ biosynthesis trait diversity. To eliminate the potential impact on the whole prokaryotic community and confirm the importance of B_12_ biosynthesis traits, linear regression analysis was also carried out between the whole prokaryotic microbial community and chlorophyll *a* concentration. Both the taxonomic profiles and functional profiles (KEGG orthologous groups) were analyzed. The analyses were carried out for samples in different layers. Spearman's rank coefficient of correlation was calculated. Correlation coefficients with significance *P* < 0.005 were termed as significant correlation.

In addition to linear regression analyses, the machine learning approach random forest was also employed to verify the importance of B_12_ biosynthesis traits on chlorophyll *a* concentration by predicting chlorophyll *a* concentrations using the functional and taxonomic profiles of B_12_ biosynthesis traits. In this study, half of the microbial data from epipelagic zones were randomly selected for developing a random forest training model, which was used to predict chlorophyll *a* concentration using the remaining microbial data in epipelagic zones. In addition, individual layers were validated, using samples from one layer (SRF/DCM) as the training set to predict the chlorophyll *a* concentration in the other layer. The relationship between predicted and observed chlorophyll *a* concentration was analyzed to evaluate the importance of B_12_ communities. The random forest analysis was performed using the “randomForest” package^68^ in R.

### Community assembly mechanisms

The null model analysis was employed to investigate the potential ecological mechanisms governing the compositional variations of B_12_ biosynthesis traits. Since the taxonomic and functional trait profiles for B_12_ biosynthesis genes were obtained by extracting targeted sequences from the shotgun metagenomic data set, phylogenetic markers for these profiles were not applicable. Therefore, the approach proposed by Zhou et al. was employed in this study^38^, ^69^. In the analysis, stochastic strength was calculated via null models to characterize the relative importance of deterministic and stochastic processes in driving the assembly of B_12_ biosynthesis traits. The within‐sample (local) and across‐sample (regional) richness were constrained to produce null models, to rule out the potential influence of local and regional species richness on beta diversity^70^. A dissimilarity matrix was calculated based on the Bray–Curtis index. The complementary similarity matrix was obtained by (1−dissimilarity). This procedure was repeated 1000 times to generate a total of 1000 null models, based on which an average similarity matrix was obtained. Community assembly stochasticity was estimated by comparing the observed and randomized community similarity, according to a modified method as described previously^53^, ^71^. The stochastic ratio was calculated considering two scenarios: (i) communities are governed by deterministic factors that produce more similar communities. In such a case, the observed community similarity ($C_{\mathrm{ij}}$) between the *i*‐th and *j*‐th communities would be larger than the null expectations ($\bar{E_{\mathrm{ij}}}$). (ii) Communities are governed by deterministic factors making communities more dissimilar. As such, $C_{\mathrm{ij}}$ would be smaller than $\bar{E_{\mathrm{ij}}}$. As a result, the observed dissimilarity ($D_{\mathrm{ij}}=1-C_{\mathrm{ij}}$) would be larger than the null model dissimilarity ($\bar{G_{\mathrm{ij}}}=1-\bar{E_{\mathrm{ij}}}$). Hence, the following functions can be used to evaluate the stochastic ratio:

$${\mathrm{ST}}_{\mathrm{ij}}^{A}=\frac{\bar{E_{\mathrm{ij}}}}{C_{\mathrm{ij}}}\;\cdot \;\frac{D_{\mathrm{ij}}}{\bar{G_{\mathrm{ij}}}}\;\text{if}\;C_{\mathrm{ij}}\geq \bar{E_{\mathrm{ij}}}$$

$${\mathrm{ST}}_{\mathrm{ij}}^{B}=\frac{\bar{G_{\mathrm{ij}}}}{D_{\mathrm{ij}}}\;\cdot \;\frac{C_{\mathrm{ij}}}{\bar{E_{\mathrm{ij}}}}\;\text{if}\;C_{\mathrm{ij}}<\bar{E_{\mathrm{ij}}}$$

$$\mathrm{ST}=\frac{\sum_{\mathrm{ij}}^{n^{A}}{\mathrm{ST}}_{\mathrm{ij}}^{A}+\sum_{\mathrm{ij}}^{n^{B}}{\mathrm{ST}}_{\mathrm{ij}}^{B}}{n^{A}+n^{B}}$$

The null model analysis was carried out for both taxonomic and functional profiles. R packages including vegan^66^, bioenv^72^, and NST^38^ were used in the analysis.

## AUTHOR CONTRIBUTIONS

**Jiayin Zhou**: Formal analysis (lead); investigation (equal); visualization (lead); writing—original draft (lead). **Wei Qin**: Conceptualization (supporting); formal analysis (supporting); writing—review and editing (supporting). **Xinda Lu**: Conceptualization (supporting); writing—review and editing (supporting). **Yunfeng Yang**: Writing—review and editing (supporting). **David Stahl**: Conceptualization (supporting); writing—review and editing (supporting). **Nianzhi Jiao**: Writing—review and editing (supporting). **Jizhong Zhou**: Conceptualization (supporting); writing—review and editing (supporting). **Jihua Liu**: Writing—review and editing (supporting). **Qichao Tu**: Conceptualization (lead); formal analysis (equal); funding acquisition (lead); writing—review and editing (lead).

## ETHICS STATEMENT

This study does not contain any studies with human participants or animals performed by any of the authors.

## CONFLICT OF INTERESTS

The authors declare no conflict of interests.

## Supporting information

Supplemental information.

Supplemental information.

Supplemental information.

Supplemental information.

## Data Availability

Sequences belonging to the B_12_ biosynthesis traits extracted from the *Tara* Oceans shotgun metagenome datasets are deposited at the ZENODO website under the record number 7520550.
